# Editorial

**Published:** 1872-07-15

**Authors:** 


MEDICAL EXAMINER.

*A Semi-Monthly Journal of Medical Sciences.*

EDITED BY

N. S. DAVIS, M. D., AND F. H. DAVIS, M. D.

Chicago, July 15th, 1872.

### EDITORIAL.

Clinical Instruction.—At the commencement of the next annual course of instruction in the Chicago Medical College, Medical Department of North-Western University, the regular system of Clinical Instruction in the Mercy Hospital and Free Dispensary will be extended in such a manner as to greatly increase the advantages of the students. The daily general clinics in the medical and surgical departments of the Mercy Hospital will be given as usual; but in addition there have been organized in connection with the Dispensary, three daily special clinics, designed for classes of students numbering not more than six or seven each. One of these special clinics will be surgical, embracing diseases of the eye and ear; another gynecological; and the other medical, embracing diseases of the chest and air-passages, with physical diagnosis. The whole clinical class will be divided into these small sub-divisions, and each afforded an oppertunity to attend a proper period of time, the special clinics in succession, thereby enabling every member of the general clinical class to have that individual training in all the details of the examination of patients and the use of instruments which cannot be obtained in large classes. The close connection of the College Faculty with the Hospital and Dispensary, and the location of these institutions upon the same lot with the college, enables them to perfect a system of clinical instruction, embracing important details that cannot be obtained in any of the schools, either in the Atlantic cities of this country or in Europe, without paying extra fees for special courses of instruction.

A Card.—For the information of the profession, the undersigned would state, that from this date he will take charge of no more patients as an attending physician, but will confine himself entirely to office and consultation practice.

As the first half of the day, from 8 A.M. to 1 P.M., is spent in the office, it is desirable that, as far as practicable, all messages desiring consultation out of the office, should be sent within the hours named, as they can during that time be seen at once and properly answered.

N. S. Davis,

Aug. 1, 1872. 797 Wabash Ave.

